# Quality of life, function and disability in individuals with chronic ankle symptoms: a cross-sectional online survey

**DOI:** 10.1186/s13047-020-00432-w

**Published:** 2020-11-16

**Authors:** M. M. Al Mahrouqi, D. A. MacDonald, B. Vicenzino, M. D. Smith

**Affiliations:** 1 Division of Physiotherapy, School of Health and Rehabilitation Sciences, The University of Queensland, St Lucia, QLD 4072 Australia; 2 Division of Physiotherapy, Oman College of Health Sciences, Ministry of Health, Muscat, P.O. Box 3720, PC 112 Sultanate of Oman; 3 Physiotherapy, School of Allied Health Sciences, Griffith University, Gold Coast, QLD 4222 Australia

**Keywords:** Ankle OA, Survey and questionnaire, Disability, Function, Quality of life

## Abstract

**Background:**

Chronic ankle conditions affect approximately 20% of Australian adults. Although there is a plethora of research on chronic hip and knee conditions, there is limited understanding of the impact of ankle problems. Thus, the significance of chronic ankle conditions is not clear. The aim of this study was to compare self-reported function, disability, instability, physical activity and quality of life (QoL) between adults with and without ankle symptoms. A secondary aim was to explore factors associated with QoL.

**Method:**

Individuals with symptoms of ankle pain and stiffness (symptomatic individuals) and controls with no ankle pain or stiffness (asymptomatic individuals) completed a cross-sectional online survey. The survey included the Ankle Osteoarthritis Scale (AOS), Foot and Ankle Ability Measure (FAAM), Cumberland Ankle Instability Tool (CAIT), International Physical Activity Questionnaire (IPAQ), Assessment of QoL (AQoL-6D), and questions about ankle injury history.

**Results:**

A total of 394 individuals (270 symptomatic and 124 asymptomatic) with mean age of 48.8 (standard deviation (SD): 12.1) years and body mass index of 28.7 (7.7) kgm^− 2^ completed the survey. Standardized mean differences (SMD) were large to very large (1.45 to 3.20) for greater disability (AOS) and instability (CAIT), and poorer function (FAAM) in symptomatic compared to asymptomatic individuals. Individuals with ankle symptoms had higher body mass index and lower QoL (medium effect: SMD > 1). There were no differences in self-report physical activity between groups. Lower activities of daily living (ADL) function (FAAM-ADL) best explained QoL in a multiple regression model (*R*^*2*^ = 0.66, p = 0.001).

**Conclusion:**

Individuals with ankle symptoms reported ankle instability, greater disability, compromised function and worse QoL compared to asymptomatic individuals. There was a strong relationship between ankle function and QoL. Ankle-specific ability during ADL best explained the reduced QoL in individuals with ankle symptoms. Clinicians and researchers should consider ankle function as an antecedent to poorer QoL in patients who have ankle symptoms.

**Supplementary information:**

**Supplementary information** accompanies this paper at 10.1186/s13047-020-00432-w.

## Background

Musculoskeletal conditions represent the second leading cause of disability affecting 20–33% of individuals worldwide [1]. Chronic ankle symptoms are a common musculoskeletal concern with an estimated prevalence of 9–20% in the adult population [2]. Persistent pain, chronic ankle instability and ankle osteoarthritis (OA) are consequences of ankle sprains and fractures, which are among the most common injuries sustained in sporting populations [3]. In light of ankle trauma occurring early in an individual’s life, chronic ankle problems are particularly important to investigate [3]. While it has been generally established that pain and physical impairments related to musculoskeletal conditions negatively impact function, mental health and quality of life (QoL) [4], there is little evidence surrounding the impact of chronic ankle problems. Saltzman et al. [5] specifically investigated individuals with severe radiographic ankle OA (Kellgren-Lawrence grade 3–4) who presented to an orthopaedic surgeon and identified that these individuals have high pain and disability and poor QoL. This raises the question of the levels of pain, disability and QoL generally in individuals who have chronic ankle problems.

Consistent with the biopsychosocial approach to management of individuals with chronic pain [6], the physical, psychological, and social aspects of chronic ankle problems should be considered in management of this population. As a first step, the relationship between the clinical presentation of individuals with chronic ankle symptoms and their self-reported pain, disability, function and QoL requires investigation. Thus, the aim of this study was to compare self-reported pain, function, ankle instability, physical activity, and QoL between individuals with chronic ankle symptoms and asymptomatic controls. A secondary aim was to identify which of these outcome measures are most associated with QoL in individuals with chronic ankle symptoms.

## Methods

An online survey of individuals with and without chronic ankle pain and/or stiffness was used to address the following questions: (i) what are the self-reported differences in QoL, function, ankle instability and physical activity between individuals with ankle symptoms and asymptomatic controls, and (ii) which of these outcome measures are associated with QoL.

### Recruitment

Between July 2015 and February 2017, Australian volunteers aged 30 to 75 years with and without a history of ankle pain and/or stiffness (present on most days for > 3 months duration) participated in this cross-sectional survey. Participants were recruited via community advertisements placed in a local university staff and community newsletters, communications from National and State arthritis organisations, and social media. Participants were asked to indicate if they “experienced any of the following ankle symptoms for more than 3 months on most days”: 1) Pain or ache in/or around the ankle, 2) Ankle joint stiffness or reduced movement in the morning. Participants who answered “yes” to either of those questions were included in the symptomatic group. Participants who indicated they did not experience any ankle pain or stiffness in the last 3 months were included in the asymptomatic control group. Exclusion criteria for control participants were a history of ankle pain or injury. The study was approved by the institutional human research ethics committee and all participants provided informed consent.

### Outcome measures

Participants provided information about their age, sex, body mass, height and history of ankle injuries and ankle related health-care consultations. They also completed the questionnaires and scales described below.

### Severity of pain and stiffness

Participants rated their ankle pain at rest, average ankle pain over the past 24-h, and worst pain over the past 7 days using an 11-point scale Numerical Rating Scale (NRS) anchored at 0 with “no pain” and at 10 with “worst pain imaginable”. Participants also rated their usual level of ankle stiffness over the past week on an 11-point NRS anchored at 0 with “no stiffness” and at 10 with “worst stiffness imaginable”.

### Quality of life

The Assessment of Quality of Life questionnaire (AQoL-6D) is an Australian multi-attribute utility instrument used to evaluate QoL with age- and gender-based population norms [7]. It comprises 20 questions in 6 separate dimensions (independent living, mental health, coping, relationships, pain, and senses). The unweighted responses of all questions are summed to create an overall profile score (0–100) and individual scores for each of the six dimensions. Higher scores indicate better QoL. This instrument has strong construct [8] and discriminative validity for use in OA populations [9].

### Function

The Foot and Ankle Ability Measure (FAAM) was used to assess function [10]. It consists of a 21-item Activities of Daily Living subscale (FAAM-ADL) and an 8-item Sports subscale (FAAM-sport). Each item is scored on a 5-point Likert scale (0–4) ranging from “no difficulty” (4) to “unable to do” (0). A “not applicable (NA)” option is available to indicate activities limited by factors other than foot or ankle problems. These items are excluded from scoring. Responses for rated items are summed, and the total scores for the FAAM-ADL and FAAM-Sport are presented as percentages, with a higher percentage indicating a higher level of function. The FAAM-ADL and Sport have excellent test-retest reliability and internal consistency [10]. At the end of the FAAM, participants rated the current level of function as normal, nearly normal, abnormal or severly abnormal.

### Pain and disability

The Ankle Osteoarthritis Scale (AOS) is a disease-specific instrument used to evaluate pain and disability related to ankle OA. It consists of pain and disability subscales, with nine questions in each subscale. Participants indicate how much pain or difficulty they experience when performing certain activities over the past week. The original scoring of the two subscales is measured along a 100-mm visual analoge scale (VAS) anchored with “No pain” or “No difficulty” at 0 mm and “Worst pain imaginable” or “So difficult, unable” at 100 mm). To enable this questionnaire to be used in an online format, an 11-point (0–10) NRS was used rather than a 100 mm VAS, with the same anchors as the original scale (paper version). To assess if the online NRS version of the questionnaire captured the same measure as the paper VAS version, paper based and online versions were administered in random order to 10 participants with ankle pain approximately 3 days apart.

### Ankle instability

The Cumberland Ankle Instability Tool (CAIT) is a valid and reliable tool used to measure perceived ankle instability [11]. The tool contains nine items with scores assigned based on the rank of the chosen response. Responses are summed separately for each limb. The maximum score is 30 with a higher score indicating less instability.

### Physical activity

The International Physical Activity Questionnaire- short form (IPAQ) was used to capture data on self-reported physical activity. The IPAQ measures the total amount of time spent performing moderate activity, vigorous activity, walking or sitting in bouts of 10 min or greater over the last 7 days [12]. The time (in minutes) spent for each activity is multiplied by the defined metabolic equivalent (MET) of each task category and scores are combined and presented as total MET-minutes per week. The IPAQ categories physical activity into “low”, “moderate” or “high”. Published guidelines for data processing and analysis of IPAQ data were used (available from: http://www.ipaq.ki.se). The IPAQ has high reliability (Spearman’s rho ranging from 0.66 to 0.88) [12].

### Statistical analysis

Statistical analysis was performed using IBM SPSS Statistics for Windows (Version 25.0. Armonk, NY: IBM Corp). Kappa statistics were used to compare the online and paper based versions of the AOS, and agreement was categorized as poor (< 0.00), slight (0.00–0.2), fair (0.21–0.4), moderate (0.41–0.6), substantial (0.61–0.8) or almost perfect (0.81–1.0) [13].

A univariate analysis of covariance with age, sex and BMI entered as covariates and group as a fixed factor was used to compare between group differences for all outcomes. To ensure our asymptomatic participants reflected the Australian population, AQoL-6D data was compared between controls and published norms. Data representing point estimates of effect are presented as mean difference (MD) and their 95% confidence intervals (CI) in tabular format and as standardized mean difference (SMD) and (CI) in forest plots. The SMD was calculated as the difference between the two group means divided by the pooled standard deviation (SD). Differences in outcomes were calculated such that negative differences indicated a deficit in the measure for the symptomatic group compared to controls, with positive differences indicating the opposite. Effect sizes were interpreted as trivial: 0.0–0.2, small: 0.2–0.6, medium: 0.6–1.2, large: 1.2–2.0, very large: 2.0–4.0 and distinct: > 4.0 [14]. Chi-square tests were conducted to compare categorical variables (sex and categories of physical activity) between groups. Odds ratio (OR) and risk difference (RD) were reported for categorical and binary data.

As bivariate normality was not assumed, the relationship between variables (AQoL-6D, group, sex, BMI, age, ankle stiffness, CAIT, AOS-Pain, FAAM-Sport, FAAM-ADL and AOS-Disability) was investigated using nonparametric Spearman’s Rank-Order Correlation. The correlation was interpreted as low (0.1 to 0.3), moderate (0.3 to 0.5), high (0.5–0.7) and very high (0.7–0.9) [14]. A stepwise backward elimination regression was conducted to establish the most influential independent variables associated with the dependent variable of AQoL-6D. The independent variables included in the model were group, sex, BMI, age, ankle stiffness, CAIT, AOS-pain, AOS-disability, FAAM-ADL and FAAM-sport. Those with a higher correlation to AQoL-6D were entered first. The multiple regression model was tested for multicollinearity. If multicollinearity was present, we retained in the model the variable with the higher β value and that has been more commonly used in research of individuals with ankle problems [15]. Statistical significance was set at *p* <  0.05.

## Results

A total of 1948 individuals responded to study advertisements of which 873 volunteers completed the online survey. After excluding individuals who did not meet the selection critiera (*n* = 298), asymptomatic respondents with a history of ankle injury or pain (*n* = 166) and removing duplicate entries (*n* = 15), survey data was available from 394 participants (263 female, mean age: 48.8 years (SD = 12.1, range = 30 to 75 years), BMI: 28.7 (SD = 7.68, range = 17.4 to 74.3). The cohort consisted of 270 participants reporting pain symptoms and 124 reporting no ankle symptoms. The majority of symptomatic participants reported both ankle pain and stiffness (93%), while a few reported either ankle pain/ache (5.9%) or stiffness (1.1%) alone in the previous 3 months. Most symptomatic individuals (92.6%) had sought help from a healthcare practitioner for their ankle symptoms (Table 1).

**Table 1:** Characteristics (symptoms, injury history and health care consultation) of the symptomatic group

Characteristic	Symptomatic
Pain intensity at rest, mean (SD)	2.91 (2.27)
Pain intensity at worst, mean (SD)	6.52 (2.42)
Average pain intensity, mean (SD)	4.62 (2.36)
Usual level of stiffness, mean (SD)	4.41 (2.68)
Unilateral ankle pain, n (%)	167 (61.9%)
Bilateral ankle pain, n (%)	100 (37%)
Unilateral ankle stiffness, n (%)	164 (60.7%)
Bilateral ankle stiffness, n (%)	90 (33.3%)
Previous injury, n (%)	
No ankle sprain	73 (27%)
Single ankle sprain	35 (13%)
Multiple ankle sprains	162 (60%)
**Previous fracture, n (%)**	
No fracture	186 (68.9%)
Single fracture	53 (19.6%)
Multiple fractures	31 (11.5%)
**Healthcare practitioner consultation for ankle, n (%)**	
General practitioner GP	182 (33.2%)
Orthopaedic surgeon	114 (20.8%)
Rheumatologist	26 (4.7%)
Sports physician	35 (6.4%)
Physiotherapist	136 (24.8%)
Osteopath	15 (2.7%)
Not visited a healthcare practitioner	40 (7.3%)

Comparison of AQoL-6D outcomes between asymptomatic controls and age and sex matched population norms [7] revealed no significant differences for any outcomes, except female controls between 55 and 64 years of age in our study had higher AQoL-6D outcomes than normative data (Additional file 1). Analysis indicated that the online NRS version of the questionnaire captured the same measure as the paper VAS version (almost perfect agreement: (0.898, 95% CI: 0.86, 0.92).

### Symptomatic ankle problems compared to asymptomatic controls

**Fig. 1:**
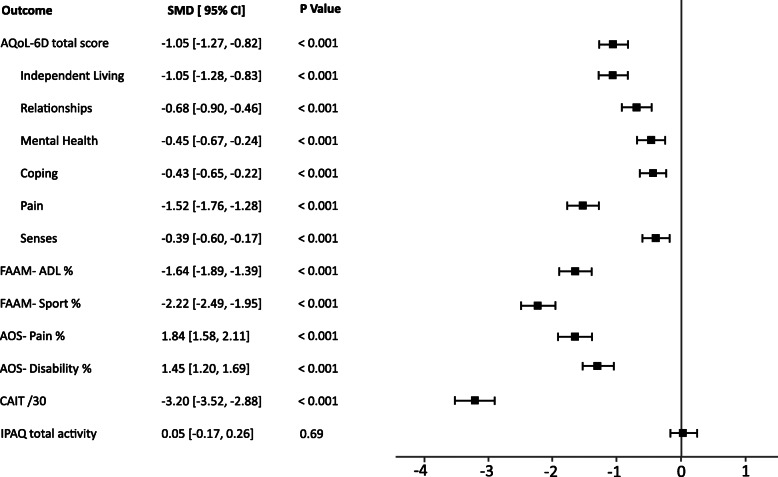
Forest plot representing the standard mean differences (SMD) and 95% confidence intervals (CI) between symptomatic and controls. Negative values indicate worse outcomes in the symptomatic group and positive values indicate worse outcome in the control group

Differences between individuals with symptomatic ankle problems and asymptomatic controls are presented in Table 1 and Fig. 1. The symptomatic ankle group were similar in age to the asymptomatic group, but had a higher BMI (SMD (95% CI) = 1 (0.74, 1.19)) and 22% fewer females (Table 2). There were large to very large differences in AOS, FAAM and CAIT outcomes (all *p* <  0.001; SMD: 1.45 to 3.2; Fig. 1) between symptomatic and asymptomatic groups. There was a medium effect for poorer total quality of life (AQoL-6D) in symptomatic compared to asymptomatic participants (*p* <  0.001; SMD: − 1.05). Total self-reported physical activity (IPAQ) was not different between groups (*p* = 0.69). For the FAAM question that asked participants to rate their level of function on a 4-point Likert scale, asymptomatic participants were more likely to rate their function as “normal” (RD 90%) [85, 95], and symptomatic participants were more likely to rate their level of function as “abnormal” or “severely abnormal” (47 and 10.7% respectively). No asymptomatic controls rated their function as abnormal or severely abnormal.

**Table 2:** Comparison of participant characteristics and outcomes between symptomatic (*n* = 270) and asymptomatic control (*n* = 124) groups

Characteristic	Symptomatic	Asymptomatic controls	MD (95%CI)	***p*** value
Age, years	48.4 (11.9, 270)	49.9 (12.3, 124)	1.5 [−1.1, 4.1]	0.25
Sex, Female n (%)	162 (60%)	101 (81.5%)	0.34 [0.20, 0.57] ^a^	**< 0.001**
BMI, kg/m^2^	30.9 (8.1, 266)	24.1 (3.9, 124)	−6.8 [− 8.0, − 5.6]	**< 0.001**
AQoL-6D, /100				
Total	72.3 (11.8, 263)	84.9 (12.4, 124)	−12.6 [− 15.4, − 9.9]	**< 0.001**
Independent Living	76.9 (15.8, 263)	93.8 (16.5, 124)	−16.9 [−20.6, − 13.3]	**< 0.001**
Relationships	81.3 (16.1, 263)	92.4 (16.8, 124)	−11.1 [− 14.8, −7.3]	**< 0.001**
Mental Health	69.5 (17.0, 263)	77.3 (17.8, 124)	−7.7 [− 11.7, −3.8]	**< 0.001**
Coping	67.1 (16.4, 263)	74.3 (17.1, 124)	−7.2 [−11.0, −3.4]	**< 0.001**
Pain	54.1 (20.5, 263)	85.8 (21.4, 124)	−31.8 [− 36.5, −27.0]	**< 0.001**
Senses	81.0 (10.9, 263)	85.3 (11.4, 124)	−4.3 [−6.8, −1.8]	**0.001**
FAAM- ADL, %	67.7 (16.9, 266)	95.9 (17.7, 113)	−28.2 [− 32.2, −24.1]	**< 0.001**
FAAM- Sport, %	48.5 (21.0, 266)	95.9 (22.0, 111)	−47.4 [−52.4, − 42.3]	**< 0.001**
FAAM-level of function, n (%)				
Severely abnormal	29 (10.7%)	0 (0%)	11% [7, 15] ^b^	
Abnormal	127 (47.0%)	0 (0%)	47% [41, 53] ^b^	
Nearly normal	106 (39.3%)	9 (7.3%)	32% [25, 39] ^b^	
Normal	8 (3.0%)	115 (92.7%)	−90% [− 95, −85] ^b^	
AOS- Overall, %	37.4 (19.4, 266)	3.9 (20.4, 108)	33.5 [28.8, 38.2]	**< 0.001**
AOS- Pain, %	38.0 (18.7, 266)	3.0 (19.6, 99)	35.0 [30.3, 39.6]	**< 0.001**
AOS- Disability, %	37.1 (22.1, 266)	4.6 (23.2, 108)	32.5 [27.2, 37.9]	**< 0.001**
CAIT, /30	10.4 (5.7, 229)	28.9 (5.9, 124)	−18.5 [−19.8, −17.1]	**< 0.001**
IPAQ total activity, MET-min/week	3417.4 (3339.5, 265)	3259.3 (3492.7, 124)	158.0 [− 616.8, 932.9]	0.69
IPAQ, level of activity, n (%)				
High	131 (48.5%)	61 (49.2%)	0% [−10, 11] ^b^	
Moderate	79 (29.3%)	48 (38.7%)	−9% [−19, 1] ^b^	
Low	59 (21.9%)	15 (12.1%)	10% [3, 18] ^b^	

^a^ Odds ratio, ^b^ Risk difference.

Abbreviations: *n* Number; *BMI* Body mass index; *SD* Standard deviation; *MD* Mean difference; *CI* Confidence interval; *p* P value/significance level; *AOS* Ankle osteoarthritis scale; *FAAM* Foot and Ankle Ability Measure; *AQoL-6D* The Assessment of Quality of Life questionnaire-6D; *CAIT* The Cumberland Ankle Instability Tool; *IPAQ* The International Physical Activity Questionnaire;

All outcomes adjusted for age, sex and BMI

Significant difference at (*p* < 0.05) based on ANCOVA post-hoc comparisons with Bonferroni correction or Pearson’s Chi-squared.

Data presented as group mean (SD, n) and MD (CI), unless otherwise stated.

### Outcomes associated with quality of life

The bivariate correlations between different survey variables are presented in Table 3. Variables were entered into the multiple linear regression model in the following order: sex, age, ankle stiffness, group, BMI, CAIT, FAAM-Sport, AOS-pain, AOS-Disability, FAAM-ADL. There was evidence of multicollinearity when including both FAAM-ADL and AOS-Disability in the model (variance inflation factors ~ 10). Initial multiple regression revealed that FAAM-ADL had the greatest contribution to AQoL-6D (β = − 0.520, *p* <  0.001) compared with the AOS-Disability (β = − 0.314, *p* = 0.001). Both of these variables explained 66.9% of the total variance. A multiple regression was re-run after removing AOS-Disability (based on β values and common use of variables in studies of individuals with ankle problems) [15].

**Table 3:** Nonparametric (Spearman’s rho) Correlations between variables

***Correlation*** ***Coefficient***	***AQoL-Total score***	***FAAM- ADL***	***AOS-Disability***	***AOS-Pain***	***FAAM-Sport***	***CAIT***	***Group***	***BMI***	***Ankle stiffness***	***Age***
***FAAM- ADL***	*0.795* *(p < 0.001, n = 380)*									
***AOS-Disability***	−0.793** *(p < 0.001,n = 375)*	− 0.942** *(p < 0.001,n = 371*								
***AOS-Pain***	−0.756** *(p < 0.001,n = 366)*	−0.899** *(p < 0.001,n = 364)*	0.923** *(p < 0.001,n = 369)*							
***FAAM-Sport***	0.738** *(p < 0.001, n = 378)*	0.926** *(p < 0.001,n = 378)*	−0.905** *(p < 0.001,n = 372)*	− 0.828** *(p < 0.001,n = 365)*						
***CAIT***	0.714** *(p < 0.001, n = 356)*	0.865** *(p < 0.001,n = 346)*	−0.842** *(p < 0.001, n = 341)*	−0.810** *(p < 0.001, n = 332)*	0.855** *(p < 0.001, n = 344)*					
***Group***	−0.409** *(p < 0.001, n = 391)*	−0.551** *(p < 0.001, n = 383)*	0.539** *(p < 0.001, n = 378)*	0.552** *(p < 0.001, n = 369*	−0.548** *(p < 0.001, n = 381)*	− 0.689** *(p < 0.001,n = 357)*				
***BMI***	−0.464** *(p < 0.001, n = 387)*	−0.507** *(p < 0.001,n = 379)*	0.514** *(p < 0.001, n = 374)*	0.459** *(p < 0.001, n = 365)*	−0.476** *(p < 0.001,n = 377)*	− 0.506** *(p < 0.001, n = 353)*	0.368** *(p < 0.001,n = 390)*			
***Ankle stiffness***	−0.404** *(p < 0.001, n = 266)*	−0.489** *(p < 0.001,n = 269)*	0.497** *(p < 0.001, n = 269)*	0.498** *(p < 0.001, n = 269)*	−0.405** *(p < 0.001,n = 269)*	−0.267** *(p < 0.001, n = 232)*	− 0.233** *(p < 0.001,n = 269)*	0.136* *(p = 0.03,n = 265)*		
***Age***	*−0.10* *(p = 0.05, n = 391)*	−0.144** *(p = 0.01,n = 383)*	0.108* *(p = 0.04,n = 378)*	*0.10* *(p = 0.07,n = 369)*	*−0.09* *(p = 0.07,n = 381)*	*−0.03* *(p = 0.63,n = 357)*	− 0.145** *(p < 0.001,n = 394)*	*0.06* *(p = 0.22,n = 390)*	0.123* *(p = 0.04,n = 269)*	
***Sex***	*0.06* *(p = 0.26,n = 391)*	0.137** *(p = 0.01, n = 383)*	*−0.09* *(p = 0.07,n = 378)*	0.133* *(p = 0.01,n = 369)*	0.129* *(p = 0.01,n = 381)*	0.114* *(p = 0.03, n = 357)*	−0.197** *(p < 0.001,n = 394)*	*−0.10* *(p = 0.06,n = 390*	*− 0.04* *(p = 0.57,n = 269)*	0.125* *(p = 0.01,n = 394)*

** Correlation is significant at the 0.01 level (2-tailed)

* Correlation is significant at the 0.05 level (2-tailed)

Abbreviations: *p* value/significance level; *AOS* Ankle osteoarthritis scale; *FAAM* Foot and Ankle Ability Measure; *AQoL-6D* The Assessment of Quality of Life; *BMI* Body mass index; *CAIT* The Cumberland Ankle Instability Tool

The most important single factor independently associated with QoL was the FAAM-ADL. It accounted for the largest amount of variance in the regression model, which explained 65.7% of the total variance (Table 4).

**Table 4:** Multiple linear regression model with the quality of life (AQoL-6D) as the dependent variable

Variables retained in the model	Standardized β weight	***P*** value	R^**2**^
FAAM-ADL	0.819	< 0.001	0.657
Age	0.067	0.087	
**Variables not retained in the model**			**Change in R**^**2**^
Ankle stiffness	0.021	0.732	0.00
FAAM-Sport	−0.055	0.583	0.00
AOS-Pain	−0.085	0.301	−0.002
Sex	−0.044	0.265	−0.002
BMI	−0.062	0.159	−0.003
Group	0.089	0.138	−0.003
CAIT	0.060	0.372	−0.001

Abbreviations: *p*-value/significance level; *AOS* Ankle osteoarthritis scale; *FAAM* Foot and Ankle Ability Measure; *ADL* Actvitiesof daily living; *AQoL-6D* The Assessment of Quality of Life; *BMI* Body mass index; *CAIT* The Cumberland Ankle Instability Tool

## Discussion

This survey compared self-reported function, disability, instability, physical activity and QoL between individuals with and without chronic ankle symptoms and investigated the associated between these factors and QoL. Our data indicate that individuals with chronic ankle symptoms reported higher BMI and disability and lower QoL, function and ankle stability than asymptomatic controls. Function during ADL was shown to be a good representation of QoL in this population. This suggests that FAAM-ADL scores could be considered as an outcome measure to determine the effectiveness of ankle management on ADL ability and QoL in clinical practice and research.

Previous research has identified poor function and QoL in individuals with CAI [16]. That research studied young (mean age 22 years) college/university students and may not represent the range of individuals with chronic ankle symptoms. The sample in our study was recruited from the community and included individuals aged 30 to 75 years, with the mean age 48.8 years, who had ankle pain and/or stiffness associated with weight-bearing activity for at least 3 months. These characteristics are consistent with guidelines for the diagnosis of OA from the National Institute of Health and Care Excellence [17]. Although we did not have radiographic evidence of OA in our symptomatic sample, we propose that the prevalence of ankle OA in this sample is likely high. This is supported by recent work at the ankle that has identified that 94% of individuals with persistent ankle pain and stiffness also had radiographic ankle OA defined as a Kellgren and Lawrence grade of ≥2 (definite osteophytes with mild-severe joint space narrowing [18, 19]. Notwithstanding this, there is evidence that it is the patients’ lower limb symptoms and not radiographic OA that is associated with their disability [20]. It is tempting to speculate that our symptomatic population is representative of ankle OA in the community. While our findings of higher disability and worse QoL and function in individuals with chronic ankle problems than asymptomatic controls is similar to that of Saltzman et al. [5] who reported high pain and disability and poor QoL in individuals with end-stage ankle OA awaiting surgery, our sample represents a very different population. Our study provides data on function, disability, instability, physical activity and QoL in community-residing individuals with chronic ankle problems, likely representative of ankle OA, that are not awaiting surgery.

High BMI in individuals with chronic ankle symptoms was associated with higher pain and disability, poorer functional capacity and worse QoL. This finding is supported by previous research reporting lower QoL in individuals with higher BMI [21, 22]. More than 65% of symptomatic participants in our study were categorised as overweight or obese (> 24.99 kg/m^2^) [23]. Obesity is characterized by excessive adipokine expression on the surface of chondrocytes [24], synoviocytes and subchondral osteoblasts [25] which increases degradative enzymes and pro-inflammatory cytokines production [26]. Obesity also modifies the joint mechanical environment due to increased joint load, inducing cartilage damage through activation of the mechanoreceptors on chondrocytes [24]. Research has shown that weight management through exercise [27] and diet [28] improves self-reported function [29], pain [29] and QoL [28] in over-weight individuals with knee OA. These data suggest that weight loss interventions may be important in managing over-weight individuals with chronic ankle symptoms.

Despite reports of lower ADL function and higher disability in individuals with ankle symptoms in our study, the level of self-reported physical activity did not differ between symptomatic and control groups. This is similar to a previous study that reported poorer function in patients who had knee and hip OA compared to controls, yet no differences in activity levels as measured by activity monitors [30]. Previous research identified that 65% of people with musculoskeletal ankle conditions limit or modify their physical activity because of an existing ankle problem [31]. Thus, it is possible that symptomatic individuals may alter the type of physical activity performed to enable pain-free or low pain physical activity participation [30]. Conversely, it is possible that asymptomatic participants were highly sedentary, resulting in no difference in physical activity levels between groups. It must also be acknowledged that self-reported physical activity outcomes (as used in our study) are less sensitive than objective measures of recording low or moderate activity [32] and may be associated with over-reporting [33]. Further research, using activity monitors, is needed to confirm whether or not physical activity levels differ between individuals with chronic ankle symptoms and controls.

Understanding the association between the different variables assessed in our study and QoL has important implications. Our data indicate that compromised foot and ankle function related to chronic ankle symptoms contributes to poorer QoL. This is supported by data from individuals with knee difficulties 5 to 20 years post anterior cruciate ligament reconstruction which found that improved function (in the form of return to sport at the same or higher level) was related to better QoL [34]. This interplay between function and QoL suggests that it is important to monitor function as an antecedent measure in optimising QoL when managing individuals with chronic ankle symptoms. This finding may inform the selection of patient-reported outcome measures in studying treatment efficacy.

Although this study provides important information on the relationship between chronic ankle symptoms, function and QoL, and the factors that influence QoL in this population, there are limitations that must be considered. First, survey data was collected using an online platform, which limited participation to internet users. Second, we specifically asked participants to indicate their level of pain, function, and ability based on ankle symptoms. However, the participant’s rating of function and ability might also be influenced by other factors (e.g. BMI related co-morbidities of which the participant is unaware), which we did not capture on our survey. Third, we did not include specific measures of potential mediating or confounding psychological factors, such as fear avoidance or pain catastrophizing. Further research should investigate the relationship between chronic ankle symptoms and these psychological factors.

This study highlights the significant burden of chronic ankle symptoms, which negatively affects QoL and function. These data, and the strong association between ADL function and QoL, suggest that management of individuals with chronic ankle symptoms should specifically target improving function. Further, the FAAM-ADL should be considered as an outcome measures to evaluate response to management in this population.

## Supplementary information

**Additional file 1.** Forest plot comparing AQoL results between survey asymptomatic respondents and published norms.

## Data Availability

The datasets generated and/or analysed during the current study are available in The University of Queensland’s Research Data Manager and are available from the corresponding author on reasonable request.

## Abbreviations

AQoL-6D: The assessment of quality of life questionnaire-6D
BMI: Body mass index
CI: Confidence interval
FAAM: Foot and ankle ability measure
IPAQ: The international physical activity questionnaire
MD: Mean difference
OA: Osteoarthritis
SD: Standard deviation
SMD: Standardised mean difference
VAS: Visual analoge scale
