# Supplementary material for: Quality of life, function and disability in individuals with chronic ankle symptoms: a cross-sectional online survey
Source: J Foot Ankle Res. 2020 Nov 16;13:67. doi: 10.1186/s13047-020-00432-w (PMC7667748; doi:10.1186/s13047-020-00432-w)

**Additional file I** Forest plot comparing AQoL results between survey asymptomatic respondents and published norms.


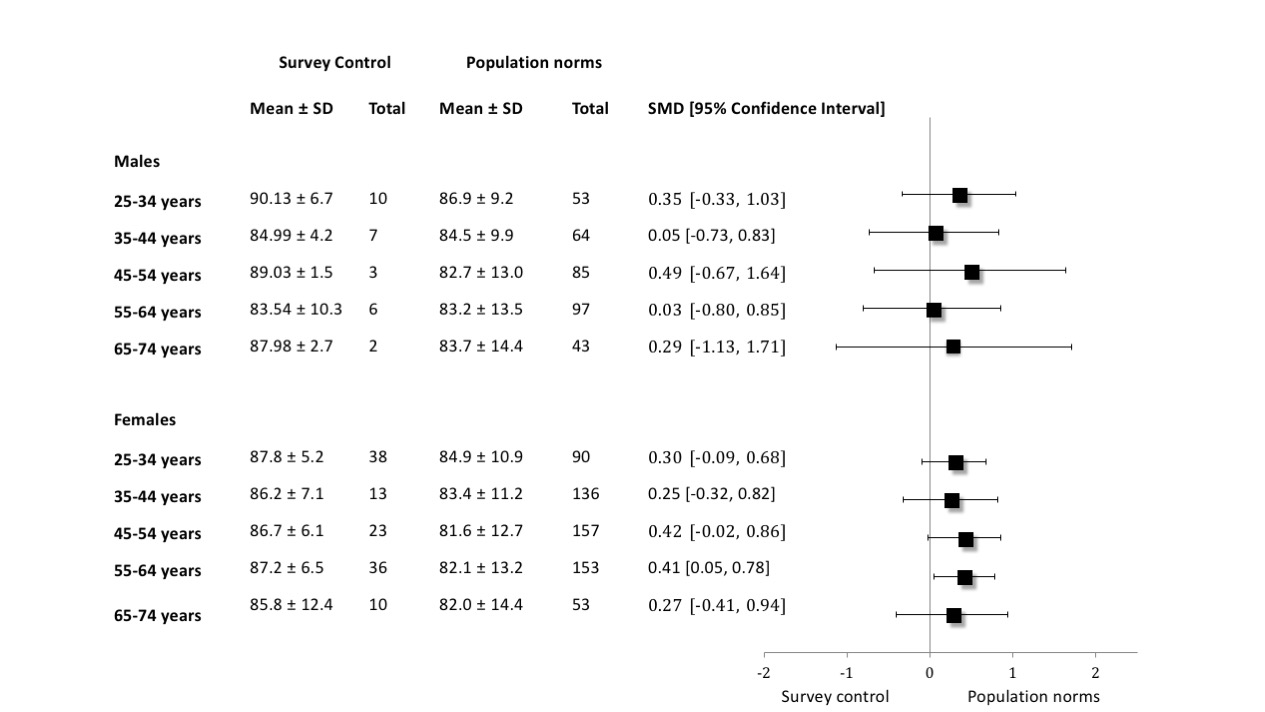

Supplement: Supplementary file 1 — Additional file 1. Forest plot comparing AQoL results between survey asymptomatic respondents and published norms. [file 13047_2020_432_MOESM1_ESM.docx]
